# NLRP3 Inflammasome Expression in Gingival Crevicular Fluid of Patients with Periodontitis and Chronic Hepatitis C

**DOI:** 10.1155/2021/6917919

**Published:** 2021-11-19

**Authors:** Petra Surlin, Luminita Lazar, Cerasella Sincar, Dorin Nicolae Gheorghe, Dora Maria Popescu, Virgil Mihail Boldeanu, Allma Pitru, Cristina Florescu, Ion Rogoveanu

**Affiliations:** ^1^Department of Periodontology, Faculty of Dental Medicine, University of Medicine and Pharmacy of Craiova, Romania; ^2^Department of Periodontology, Faculty of Dental Medicine, “George Emil Palade” University of Medicine and Pharmacy, Targu Mures, Romania; ^3^Department of Periodontology, Faculty of Medicine, “Dunarea de Jos” University, Galati, Romania; ^4^Department of Immunology, Faculty of Medicine, University of Medicine and Pharmacy of Craiova, Romania; ^5^Department of Oral Pathology, Faculty of Dental Medicine, University of Medicine and Pharmacy of Craiova, Romania; ^6^Department of Internal Medicine and Cardiology, Faculty of Medicine, University of Medicine and Pharmacy of Craiova, Romania; ^7^Department of Gastroenterology, Faculty of Medicine, University of Medicine and Pharmacy of Craiova, Romania

## Abstract

The study is aimed at assessing the impact that periodontal disease and chronic hepatitis C could have on gingival crevicular fluid levels of the NLRP3 inflammasome, caspase-1 (CASP-1), and interleukin-18 (IL-18) and at evaluating whether the increased local inflammatory reaction with clinical periodontal consequences is correlated to their upregulation. Patients were divided into four groups, according to their periodontal status and previously diagnosed hepatitis C, as follows: (i) CHC group, chronic hepatitis C patients; (ii) P group, periodontal disease patients, systemically healthy; (iii) CHC + P group, patients suffering from both conditions; and (iv) H group, systemically and periodontally healthy controls. Gingival crevicular samples were collected for quantitative analysis of the NLRP3 inflammasome, CASP-1, and IL-18. CHC + P patients expressed the worse periodontal status and the highest NLRP3, CASP-1, and IL-18 levels, the difference being statistically significant (*p* < 0.05). The P group patients also expressed significantly more elevated NLRP3, CASP-1, and IL-18 levels, as compared to nonperiodontal patients (CHC and H groups). Chronic hepatitis C and periodontal disease could have a significant influence on the upregulation of NLRP3 inflammasome and its components, possibly contributing to an increased local inflammatory reaction and clinical periodontal consequences.

## 1. Introduction

Recent research on the complex molecular mechanisms of the inflammatory reaction has led to the development of the “inflammasome” concept, a multiprotein, oligomer compound, governing inflammation in its early stages [1, 2]. One of these inflammasomes, the NLRP3 complex, has a crucial role in innate immunity and inflammatory mechanisms [3, 4]. It consists of a Nod-like receptor (NLR) that mediates the activation of protease enzymes (Caspase 1 (CASP-1)) and further regulates the expression of pioneer, key, proinflammatory cytokines, such as interleukin-18 (IL-18) [3].

The periodontal inflammatory process, which defines periodontitis (P), can interact with the chronic inflammatory reaction generated by viral hepatic infection, resulting in systemically important pathogenic implications [5]. The activation of the NLRP3 is caused by bacterial stimuli, as lipopolysaccharides (from *Porphyromonas gingivalis*) and bacterial RNA or by endogenous ones, as extracellular ATP, uric acid, or cholesterol crystals [3]. Despite its elevated concentrations within epithelial tissues, such as the gingival epithelium [6], the NLRP3 inflammasome has received little scientific attention from periodontal researchers, as part of the pathogenic mechanisms governing P alone, or in association with systemic diseases [7–9]. A recent study by Hernandez et al. highlighted the upregulation of the NLPR3 inflammasome in patients with periodontitis and uncontrolled type 2 diabetes [8]. This could also be the case in patients with periodontitis and cardiovascular diseases, as highlighted by Mahendra et al. [10]. The activation of the NLRP3 inflammasome during periodontal inflammation has been illustrated by using samples of serum and saliva, by Isola et al. [11]. Thus, the study of this particular inflammasome (NLRP3) and its components (CASP-1 and IL-18) was chosen as a focus point of our research, considering the promising results of its assessment as a potential biomarker for the periodontal clinical status [2] and its upregulation in periodontal patients with uncontrolled type 2 diabetes [6, 8, 11].

Chronic hepatitis C (CHC) patients can often manifest important oral health issues, that can have a negative impact on their life's quality, adding to the pathological manifestations of the liver disease and its complications [12]. When seeking dental treatments, CHC patients may face various elements of difficulty, such as high personal anxiety or modified healing and recovery processes after dental and periodontal surgery, that limit the complexity of therapeutical options [13]. Corroborated with possible behavioral particularities, CHC patients may comprise more risk factors for the onset of P, leading to its clinical manifestation, triggered by the accumulation of subgingival bacterial plaque deposits [5, 14].

In essence, both CHC and P generate a chronic inflammatory reaction, such a pathologic event being driven by proinflammatory mediators that control its extent and intensity [15, 16]. Our previously published study, focusing on the gingival crevicular fluid (GCF) assessment of interleukin-1alpha's and interleukin-1beta's involvement in the pathogenic process of periodontitis patients with chronic hepatitis C, highlighted a significantly worsened periodontal status and increased levels of these cytokines in patients with both diseases, as compared to those of nonhepatitis C patients suffering from periodontitis [17]. This suggests the negative impact that hepatic pathology may have on local periodontal inflammation.

Hence, the purpose of the present study was to evaluate the GCF levels of the NLRP3 inflammasome and its components in patients with P and CHC and to determine if its upregulation is influenced by these joined pathologies, periodontitis, and chronic hepatitis C, leading to a more exacerbated manifestation of periodontal disease in CHC patients.

## 2. Materials and Methods

### 2.1. Study Design

The study's design was approved by the Ethical Research Committees at the University of Medicine and Pharmacy of Craiova, Romania, and at the Craiova Emergency County Hospital fulfilling the requirements of the European Union's General Data Protection Regulation (GDPR) on patient data protection and discretion and the 1975-2013 Declaration of Helsinki. The study approached two main research directions: the first one, clinical and metabolic, consisting of assessment of the patient's periodontal status and certain metabolic parameters, which reflected their systemic and hepatic status; the second direction, immunological, consisting of a quantitative determination of the targeted inflammatory markers within the patients' gingival crevicular samples. The data resulting from the two study directions was subjected to intra- and intergroup statistical analysis for significance and correlation identification.

### 2.2. Patient Selection

Chronic hepatitis C participants were selected from patients attending the Gastroenterology Clinic of the Craiova Emergency County Hospital, while nonhepatitis C participants were selected from the patients addressing the Periodontology Department of the Dental Medicine Faculty of the University of Medicine and Pharmacy of Craiova. For inclusion, all hepatitis C patients had asymptomatic forms of disease. All periodontal patients had to meet the diagnosis criteria issued during the 1996 World Workshop in Clinical Periodontics: (i) minimum of 20 existing natural teeth, (ii) minimum of six periodontal pockets in two different quadrants (probing depth ≥ 5 mm), (iii) bleeding on probing, and (iv) minimum one mm clinical attachment loss [18]. According to the 2018 new classification system of periodontal diseases [19], this would correspond to Stage II (moderate) and Stage III (severe) periodontitis, in terms of severity and Grade A (slow) in terms of rate of progression. For inclusion in the healthy control group, patients had to show no symptoms and history of periodontal or gingival disease (no periodontal pockets/bone loss, no gingival bleeding) and no declared systemic diseases.

The exclusion criteria consisted of (i) anti-inflammatory or other type of medication taken in the last 30 days prior to examination, (ii) previous antiviral anti-HCV therapy, (iii) antibiotic treatment taken in the last 90 days prior to examination, (iv) pregnancy, (v) active smoking status, and (vi) other associated systemic diseases.

Upon applying all of the above inclusion/exclusion criteria and obtaining the informed and written individual consent for entering the study, the 62 participating patients were divided into four study groups: (i) CHC + P group: 18 patients (aged from 54 to 79 years) suffering from both CHC and P; (ii) CHC group: 14 patients (aged from 50 to 61 years), suffering only from CHC and being periodontally healthy; (iii) P group: 15 patients (aged from 42 to 63 years), suffering only from P and being systemically healthy; and (iv) H group: 15 controls (aged from 40 to 61 years), periodontally and systemically healthy patients.

### 2.3. Dental and Periodontal Assessment

All participating patients were subjected to a complete oral and periodontal examination, which was used for the diagnosis of possible periodontal conditions. During this examination, the level of dental hygiene was evaluated, by using the O'Leary Index [20].

After the assessment of the oral hygiene level, an ultrasonic scaling procedure of calculus and plaque deposits' removal was conducted when required, in order to enable unbiased and undisturbed periodontal probing. The periodontal measurements were performed by using manual University of North Carolina probes (Hu-Friedy, Chicago, Illinois, USA). The periodontal probing provided periodontal parameters such as (i) periodontal probing depth (PD)—in six points for each tooth (messial, central, and distal for the buccal and oral aspects of the teeth), (ii) clinical attachment loss (AL), and (iii) bleeding on probing index (BPI). All periodontal probing measurements were performed by a single, calibrated, examiner. The attachment loss was calculated for each probing site by using the formula: periodontal probing depth (mms)–gingival margin (mms) = attachment level (AL, mms).

### 2.4. Metabolic and Hepatic Assessment

From each patient, a blood sample was collected for the laboratory tests required for the assessment of their metabolic status. This included standard laboratory blood tests such as (i) level of serum glucose—glycemia, as indicator for the glucidic metabolism (reference range 90-110 mg/dL) [21]; (ii) level of total cholesterol, as indicator for the lipidic metabolism (reference range 140–180 mg/dL) [21]; and (iii) level of triglycerides, as a complementary indicator for lipidic metabolism (reference range 140–160 mg/dL) [21]. Specific parameters which allowed the assessment of the hepatic function were also tested (i) aspartate aminotransferase (AST) (reference range 6-34 IU/L) [22], (ii) alanine transaminase (ALT) (reference range 20-60 IU/L) [22], and (iii) gamma-glutamyl transferase (GGT) (reference range 8-38 IU/L) [22]. The hepatic status was also evaluated through the use of ultrasound elastography imaging test (FibroScan 530, Echosens, Paris, France) for the degree of liver fibrosis in CHC patients. The scale used to assess the level of liver fibrosis was from zero (= absent) to four (= liver cirrhosis) [23].

### 2.5. Gingival Crevicular Fluid Sampling

After the supragingival plaque was removed with sterile cotton bullets, gingival crevicular fluid (GCF) was sampled from each of the 62 patients, by using absorbent paper strips (PerioPaper, Oraflow Inc., Smithtown, NY, USA). The two teeth with the deepest pocket depths were selected for sampling, using different paper strips for each tooth, inserted simultaneously at the two teeth. The paper strips were inserted within the periodontal pocket until mild resistance was felt and kept in place for 30 seconds. Upon removal, the strips were visually inspected for blood stains. In order to prevent the strips' contamination with saliva or blood, absorbent cotton roll isolation and air suction were used during the sampling procedure. The quantity of sampled GCF was standardized using the Periotron 8000 device (Oraflow Inc., Smithtown, NY, USA). Afterwards, the paper strips originating from both teeth were pooled together into a plastic microtube containing saline buffer solution (PBS). The sampling procedures were additionally repeated two times. Thus, three microtubes were obtained for each patient, corresponding to the three targeted mediators. The microtubes were preserved at -20 degrees Celsius, until analysis.

### 2.6. Immunological Assessment

After the GCF samples were collected from all 62 patients, they were transferred to the Immunology Laboratory of the University of Medicine and Pharmacy of Craiova for specific preparation and assessment. For the detection of the targeted inflammatory mediators (NLRP3 inflammasome, CASP-1, and IL-18) within the gingival fluid, the enzyme-linked immunosorbent assay (ELISA) method was used. Specifically designed commercial test kits were used for each of the mediators, (i) NLRP3—OKEH03368 (Aviva Systems Biology, San Diego, USA) (range 0.312-20 ng/mL), (ii) CASP1—OKEH01146 (Aviva Systems Biology, San Diego, USA) (range 15.6-1000 pg/mL), and (iii) IL-18—OKCD00106 (Aviva Systems Biology, San Diego, USA) (range: 15.6-1000 pg/mL), according to the manufacturer's indications and prescribed work method. The ELISA method was performed with a standard optical analyzer, at 450 nm wave length.

### 2.7. Statistical Analysis

All data was centralized and expressed as mean and standard deviation. Afterwards, it was subjected to statistical analysis (GraphPad Software, LLC, San Diego, CA, USA) in order to detect the differences between patients with CHC + P and CHC, CHC + P and P, and CHC and P groups, using Mann–Whitney *U* test (statistically significant at 5%, two-tailed). For categorical variables, the comparisons between the groups were evaluated using the Fisher's exact test. The existence of statistical correlations between the different types of data was assessed using Pearson coefficients. A power computation (G∗Power 3, University of Dusseldorf, Germany) was completed and revealed that, for our samples, the effect size was large (1.468) and power equal to 0.979 (alpha equal to 0.05).

## 3. Results

### 3.1. Demographics, Glucose, and Liver Fibrosis Level

There was no statistical difference regarding the average age of the study's participants or the gender distribution among the study groups (*p* > 0.05) (Table 1). There was no significant difference concerning the level of serum glucose among the test groups. In the hepatitis C patients (groups CHC + P and CHC), there was a similar level of liver fibrosis (predominatly F1, 61.11% in the CHC + P group and 64.28% in the CHC group), with no significant difference (*p* > 0.05). The CHC + P group included significantly more patients with F2 stage liver fibrosis than the CHC group (Table 1).

### 3.2. Patients with CHC + P Expressed the Most Severely Modified Clinical Periodontal Status

The patients of the CHC + P group showed a significantly worsened periodontal status as compared to P patients, in terms of periodontal probing depth and clinical attachment loss (Table 2). A statistically significant difference was also found between the plaque index and gingival bleeding index of the CHC + P and P groups (Table 2).

### 3.3. Patients with CHC + P Exhibited Significantly Elevated Levels of Proinflammatory Mediators

The highest values of the assessed mediators were recorded for the CHC + P group, being significantly more elevated that those of the other groups (Table 3). Both groups of periodontitis patients (CHC + P and P) expressed significantly elevated levels for all of the three assessed proinflammatory mediators (NLRP3, CASP1, and IL-18) as compared to the nonperiodontitis groups (CHC and H) (Table 3). The proinflammatory mediators' levels in all three test groups were significantly more elevated than in controls (Table 3).

### 3.4. NLRP3, CASP1, and IL-18 GCF Levels Associated Positively with Certain Periodontal and Metabolic Parameters

The results delivered significant positive correlations between the GCF NLRP3 levels and the periodontal probing depth, clinical attachment loss, and gingival bleeding index (*r* = 0.4; *p* < 0.05) (Table 4). The NLRP3 GCF levels also positively correlated with certain metabolic parameters, including the glucose, aspartatetransferase (AST), and alaninetransferase (ALT) levels. Other significant positive correlations were found between the CASP1 GCF levels and the age of the patients, the clinical attachment loss, gingival bleeding index, and ALT level (Table 4). The GCF IL-18 levels significantly correlated with the age of patients, the periodontal probing depth, the clinical attachment loss, the gingival bleeding index, and triglyceride level (Table 4). No correlations were identified between the level of liver fibrosis and the assessed proinflammatory mediators.

## 4. Discussion

The development of the “inflammasome” concept has opened new perspectives for periodontal inflammation research. NLPR3 is a key element of this inflammatory reaction, being considered as its pioneer triggering factor, from the first initial contact with bacterial antigens, such as *Porphyromonas gingivalis*' lipopolysaccharides (LPS) [24–26]. While some authors state that LPS can stimulate NLRP3 synthesis by activation of the Toll-like 4 receptor [27], others have observed that the inflammasome decreases its activity when in contact with subgingival bacteria [28]. In our study, there was no correlation between the GCF NLRP3 levels and the level of bacterial plaque. However, certain studies reported that NLRP3 expression is more elevated in periodontitis patients than in healthy controls [29], both in specific cells [8], and within saliva [11, 30]. Our study delivered similar results, highlighting the significant differences of the GCF NLRP3 levels between the P and H groups and between the CHC + P and CHC ones, which suggest that periodontal pathological events trigger a considerable increase of NLRP3 expression. This hypothesis was also assessed and endorsed by a recent review on the subject, which concluded that periodontal diseases can be characterized by an upregulation of inflammasomes, alongside with a downregulation of their inhibitor proteins [29].

The statistical results of our study highlighted significant correlations between the GCF NLRP3 levels and serum glucose levels of the participating patients, in accordance with other studies on the topic of NLRP3 inflammasome, periodontal disease, and diabetes association [9, 11, 31–34]. This correlation endorses the pathogenic links existing between periodontal diseases and cellular resistance to insulin [35]. Additional pathogenic connections also lay between chronic hepatitis C and insulin resistance [36]. These elements suggest that this pathologic mechanism could bridge the connection between chronic hepatitis and periodontal disease, through the significant impact and bi-directional consequences it inflicts on the inflammatory reaction.

The immunological analysis performed in our study generated comparable outputs on the average values of CASP1 within the GCF samples of periodontitis patients, which were significantly higher than those of healthy controls [37]. Histologically, CASP1 is predominantly expressed within gingival epithelial cells, as keratinocytes, and connective tissue cells, being almost absent when the periodontal tissues are not inflamed [37–39]. Moreover, important periodontal bacterial pathogens (*Aggregatibacter actinomycetemcomitans* and *Porphyromonas gingivalis*) have been shown to trigger caspase expression within epithelial cells and macrophages [40, 41]. Within this finding, in our study also, a significant positive correlation was identified between the GCF CASP1 levels and the clinical periodontal parameters used for assessing the severity of periodontal damage (clinical attachment loss and gingival bleeding index).

Our current study's results show, as similar other ones, that the GCF IL-18 levels were significantly higher in the samples of periodontal patients as compared to healthy controls [42–45]. Moreover, the statistical analysis denoted significant correlations between the GCF IL-18 levels and the parameters of periodontal disease's severity (periodontal probing depth, clinical attachment loos, and gingival bleeding index), similar to those highlighted by other papers [29]. IL-18 has also been suggested as a possible indicator for periodontal structures' damage [45]. In accordance with this finding, our CHC + P patients expressed significantly elevated GCF IL-18, reflecting their unfavorable clinical periodontal status.

Regarding the hepatic pathology, NLRP3 has been observed to activate when stimulated by the hepatitis C virus, within white and red blood cells [46–48]. Moreover, in an *in vitro* setting, the presence of the virus determined cellular pyroptosis within infected hepatocytes, an event mainly controlled by NLRP3 and CASP1 activity [47]. NLRP3 is also involved in the onset of the chronic hepatic inflammatory reaction, consequent to HCV infection, together with IL-1*β* [48]. Thus, CHC patients are expected to exhibit elevated serum NLRP3 levels. This fact has also been shown by the results of our study that identified more elevated GCF NLRP3 levels in the samples of CHC patients, that those of the H group, given that GCF is a blood serum derivate. As shown by the delivered results, when CHC patients also suffer from P, their GCF NLRP3 levels increased significantly compared to those of patients suffering from CHC or P alone, suggesting that NLRP3 expression is upregulated when the two diseases occur in the same patient.

CASP1 is also involved in the pathological processes of CHC, as the HCV-infected cells are able to synthesis and release the NLRP3 inflammasome [49, 50]. Our study's results highlighted more elevated average GCF CASP1 levels in the samples of CHC + P patients, as compared to P patients. Significant differences were also identified between the average values of the CHC patients, with and without periodontal disease. These results suggest the significant impact that chronic inflammation, either hepatic or periodontal, could have on the GCF CASP1 levels.

Concerning the hepatic disease, IL-18 also has immunological and clinical implications on CHC, as affected patients often exhibit significantly elevated values of this mediator, compared to healthy controls [51–54]. In our study, significant differences of the GCF IL-18 levels were recorded between the CHC + P group and the P group, endorsing the negative impact that HCV infection can have on the periodontal status, in similar periodontal pathological settings. This can amplify gingival pathogenic events and trigger a more intense and severe periodontal inflammatory reaction.

Our results show that CHC + P patients exhibited a more severely modified clinical periodontal status. In addition, these patients also exhibited the highest NLRP3 GCF levels. The negative character of their periodontal status could be in relation to the upregulation of this mediator. A study by Garcia-Hernandez et al. stated that the upregulated NLRP3 levels could increase the inflammatory response in uncontrolled type-2 diabetes periodontal patients [8]. This was also shown by a recent study by Isola et al., in samples of saliva and serum, originating from patients with periodontitis and diabetes [11]. Concerning CHC periodontal patients, future extended research is required to test this hypothesis.

Given the sample size limitations, all three assessed elements (NLRP3, CASP1, and IL-18) expressed significantly elevated GCF levels in CHC + P patients, as compared to the other groups, suggesting that the coexisting hepatic pathology might have an upregulating effect on these proinflammatory mediators. The decline of the clinical periodontal and immunological status of these patients should be taken into account when developing holistic therapeutical strategies.

## 5. Conclusions

Within the limitations of this study, we could conclude that chronic hepatitis C and periodontitis might have a joined effect on the upregulation of the NLRP3 inflammasome and its components in the GCF. This fact could justify the exacerbated manifestation of periodontal disease in chronic hepatitis C patients. This motivates future expanded research on the matter.

## Figures and Tables

**Table 1:** Study's demographic characteristics, serum glucose level, liver fibrosis, and statistical significance (*p* value) for differences between test groups.

Parameter	Groups				*p* CHC + P vs. CHC	*p* CHC + P vs. P	*p* CHC vs. P
	CHC + P	CHC	P	H			
Age (years)	62.61	57.76	55.86	50.13	> 0.05	> 0.05	> 0.05
Gender (male/female)	8/10	5/9	8/7	6/9	> 0.05	> 0.05	> 0.05
Glucose (mg/dl) ± SD	108.83 ± 27.22	103.14 ± 16.54	98.2 ± 13.43	95.53 ± 8.75	> 0.05	> 0.05	> 0.05
Liver fibrosis (%)			—	—		—	—
F1	61.11	64.28			> 0.05		
F2	22.22	7.14			< 0.05		
F3	11.11	14.28			> 0.05		
F4	5.55	14.28			> 0.05		

**Table 2:** Study's clinical results and statistical significance (*p* value) for differences between test groups.

Parameter	Groups				*p* CHC + P vs. CHC	*p* CHC + P vs. P	*p* CHC vs. P
	CHC + P	CHC	P	H			
Periodontal probing depth (mm) ± SD	3.85 ± 0.92	1.45 ± 0.2	3.76 ± 0.53	1.24 ± 0.2	< 0.05	< 0.05	< 0.05
Clinical attachment loss (mm) ± SD	5.42 ± 1.61	1.83 ± 0.42	3.98 ± 0.51	0.52 ± 0.19	< 0.05	< 0.05	< 0.05
Plaque index (%) ± SD	57.5 ± 17.27	25.85 ± 13.63	39.33 ± 12.61	13.86 ± 4.03	< 0.05	< 0.05	< 0.05
Gingival bleeding index (%) ± SD	50.53 ± 11.98	7.92 ± 5.31	43.6 ± 13.18	7.4 ± 2.35	< 0.05	< 0.05	< 0.05

**Table 3:** Study's immunological results and statistical significance (*p* value) for differences between test groups.

Parameter	Groups				*p* CHC + P vs. CHC	*p* CHC + P vs. P	*p* CHC vs. P
	CHC + P	CHC	P	H			
NLRP3 (ng/mL) ± SD	1.534 ± 0.32	0.989 ± 0.3	1.251 ± 0.36	0.583 ± 0.18	< 0.05	< 0.05	< 0.05
CASP1 (ng/mL) ± SD	0.369 ± 0.14	0.2 ± 0.07	0.283 ± 0.08	0.05 ± 0.04	< 0.05	< 0.05	< 0.05
IL − 18 (ng/mL) ± SD	0.287 ± 0.07	0.193 ± 0.05	0.2 ± 0.03	0.075 ± 0.07	< 0.05	< 0.05	< 0.05

**Table 4:** Synopsis of correlation statistical assessment (Pearson's test) for whole patient batch between NLRP3, CASP-1, and IL-18 levels and assessed parameters.

Parameter	Proinflammatory mediator		
	NRLP3	CASP1	IL-18
	*r*/*p*	*r*/*p*	*r*/*p*
Gender	0.05/0.8	0.08/0.71	0.14/0.52
Age	0.33/0.12	0.56/0.005^∗^	0.43/0.04^∗^
Periodontal probing depth	0.45/0.03^∗^	0.39/0.06	0.47/0.02^∗^
Clinical attachment loss	0.52/0.01^∗^	0.46/0.02^∗^	0.52/0.013^∗^
Plaque index	0.27/0.21	0.36/0.09	0.4/0.06
Gingival bleeding index	0.45/0.03^∗^	0.48/0.02^∗^	0.42/0.04^∗^
Glucose	0.43/0.04^∗^	0.14/0.51	0.03/0.86
Cholesterol	0.02/0.9	0.26/0.22	0.39/0.06
Triglycerides	0.23/0.28	0.05/0.79	0.42/0.04^∗^
Aspartate transaminase	0.45/0.04^∗^	0.14/0.52	0.14/0.5
Alanine transaminase	0.51/0.02^∗^	0.57/0.02^∗^	0.08/0.69
Glutamyl transferase	0.2/0.35	0.18/0.4	0.05/081

*r*: Pearson's *r*; *p*: statistical significance. ^∗^Statistically significant value (*p* < 0.05).

## Data Availability

The data used to support the findings of this study are available from the corresponding author upon reasonable request.
